# Progress and challenges in biomaterials used for bone tissue engineering: bioactive glasses and elastomeric composites

**DOI:** 10.1186/2194-0517-1-2

**Published:** 2012-09-26

**Authors:** Qizhi Chen, Chenghao Zhu, George A Thouas

**Affiliations:** 1 Department of Materials Engineering, Monash University, Clayton, Victoria 3800 Australia; 2 Department of Zoology, The University of Melbourne, Parkville, Victoria 3010 Australia

**Keywords:** Bioceramic, Elastomer, Composite, Mechanical property, Degradation

## Abstract

Driven by the increasing economic burden associated with bone injury and disease, biomaterial development for bone repair represents the most active research area in the field of tissue engineering. This article provides an update on recent advances in the development of bioactive biomaterials for bone regeneration. Special attention is paid to the recent developments of sintered Na-containing bioactive glasses, borate-based bioactive glasses, those doped with trace elements (such as Cu, Zn, and Sr), and novel elastomeric composites. Although bioactive glasses are not new to bone tissue engineering, their tunable mechanical properties, biodegradation rates, and ability to support bone and vascular tissue regeneration, as well as osteoblast differentiation from stem and progenitor cells, are superior to other bioceramics. Recent progresses on the development of borate bioactive glasses and trace element-doped bioactive glasses expand the repertoire of bioactive glasses. Although boride and other trace elements have beneficial effects on bone remodeling and/or associated angiogenesis, the risk of toxicity at high levels must be highly regarded in the design of new composition of bioactive biomaterials so that the release of these elements must be satisfactorily lower than their biologically safe levels. Elastomeric composites are superior to the more commonly used thermoplastic-matrix composites, owing to the well-defined elastic properties of elastomers which are ideal for the replacement of collagen, a key elastic protein within the bone tissue. Artificial bone matrix made from elastomeric composites can, therefore, offer both sound mechanical integrity and flexibility in the dynamic environment of injured bone.

**Electronic supplementary material:**

The online version of this article (doi:10.1186/2194-0517-1-2) contains supplementary material, which is available to authorized users.

## Introduction

Tissue engineering is ‘the application of principles and methods of engineering and life sciences to obtain a fundamental understanding of structure-function relationships in normal and pathological mammalian tissue, and the development of biological substitutes to restore, maintain, or improve tissue function’ (Skalak and Fox [1993]). A common approach is to harvest an expansion of living tissue *in vitro* and design of biomaterial scaffolds to provide appropriate structural support to match the tissue of interest. Scaffolds are then loaded with numbers of cells and numbers for implantation, which allows surgeons to manipulate local tissue environments, providing more physiological alternatives to standard approaches in reconstructive surgery (Bell [2000]).

There are several requirements of scaffold materials to meet the demands of tissue engineering. Firstly, biocompatibility of the substrate materials is imperative. The material must not elicit an unresolved inflammatory response nor demonstrate immunogenicity or cytotoxicity. As with all materials in contact with the human body, tissue scaffolds must be easily sterilizable to prevent infection (Chaikof et al. [2002]). This applies notably for bulk degradable scaffolds, where both the surface and the bulk material must be sterile. In addition, the mechanical properties of the scaffold must be sufficient to prevent structural failure during handling and during the patient's normal activities. A further requirement for a scaffold, particularly in bone engineering, is a controllable interconnected porosity that can direct cells to grow into a physical structure and to support vascularisation. A typical porosity of 90% as well as a pore diameter of at least 100 μm is known to be compulsory for cell penetration and a proper vascularization of the ingrown tissue (Griffith [2002]; Karageorgiou and Kaplan [2005]; Levenberg and Langer [2004]; Mikos and Temenoff [2000]). Other desirable aspect concerns the cost-effectiveness of scaffold processing toward industrial-scale production to reliably generate net-like structures with a nominal range of porosities.

Materials used for bone tissue engineering scaffolds include the following: (1) natural or synthetic polymers such as proteins, thermoplastics, hydrogels, thermoplastic elastomers (Berger et al. [2004]; Drotleff et al. [2004]; Mano et al. [2004]; Tirelli et al. [2002]) and chemically cross-linked elastomers (Chen et al. [2008b]), (2) bioactive ceramics such as calcium phosphates and bioactive glasses or glass ceramics (Hench [1998]; Kim et al. [2004]; Levenberg and Langer [2004]), (3) composites of polymers and ceramics (Boccaccini et al. [2005]; Hedberg et al. [2005]; Kim et al. [2004]; Niiranen et al. [2004]; Yao et al. [2005]; Zhang et al. [2004]), and (4) metallic materials such as titanium and magnesium alloys (Lefebvre et al. [2008]). From the material science point of view, bone is a natural composite of inorganic calcium phosphate apatite and biological polymers including collagens, which are deposited by residence osteocytes. The composite system of polymers and ceramics is apparently a logic choice for bone tissue engineering, as demonstrated by the huge research efforts worldwide using these materials (Boccaccini et al. [2005]; Di Silvio and Bonfield [1999]; Gittens and Uludag [2001]; Hedberg et al. [2005]; Jiang et al. [2005]; Khan et al. [2004]; Kim et al. [2004]; Li and Chang [2004]; Lu et al. [2005]; Luginbuehl et al. [2004]; Mano et al. [2004]; Maquet et al. [2004]; Niiranen et al. [2004]; Xu et al. [2004]; Yao et al. [2005]; Zhang et al. [2004]).

The present authors previously reviewed biodegradable thermoplastic polymers and bioactive ceramics, including strategies for fabrication of composite scaffolds with defined microstructure and mechanical properties, and methods of *in vitro* and *in vivo* evaluation (Rezwan et al. [2006]). Over the past 10 years, new processes of Na-containing bioactive glasses and new bioactive glass compositions doped with various trace elements have been developed aiming at healthy bone growth and/or vascularization (Rahaman et al. [2011]). Meanwhile degradable elastomeric polymers have gained increasing attentions in the field of tissue engineering, mainly because of the inherent structural elasticity of biological tissues. Composite scaffolds made from bioceramics and chemically cross-linked elastomers have proven beneficial in terms of both biocompatibility and their operation over a wide range of elastic moduli (Chen et al. [2010a]; Liang et al. [2010]). This article aims to provide an update on the progress of biomaterials developed for bone tissue engineering, with a specific focus on bioactive glasses and elastomeric composites that show potentials to advance bone tissue engineering, while the rest of biomaterials in bone tissue engineering are reviewed briefly for a complete overview.

## Biodegradable and surface erodible thermoplastic polymers

Based on their mechanical properties, polymeric biomaterials can be classified as elastomers and non-elastomeric thermoplastics. This section will provide a brief review on biodegradable thermoplastics. Comprehensive discussions of these polymers and their physical properties have been provided in great detail elsewhere (Chen and Wu [2005]; Gunatillake et al. [2003a]; Iroh [1999]; Kellomäki et al. [2000]; Kumudine and Premachandra [1999]; Lu and Mikos [1999]; Magill [1999]; Middleton and Tipton [2000]; Ramakrishna et al. [2004]; Rezwan et al. [2006]; Seal et al. [2001]; Yang et al. [2001]).

The most widely utilized biodegradable synthetic polymers for 3D scaffolds in tissue engineering are saturated aliphatic polyesters, typically poly-α-hydroxy esters including poly(lactic acid) (PLA), poly(glycolic acid) (PGA) (Gollwitzer et al. [2005]; Seal et al. [2001]), poly(ϵ-caprolactone) (PCL) (Pitt et al. [1981]), and their copolymers (Jagur-Grodzinski [1999]; Kohn and Langer [1996]; Mano et al. [2004]; Seal et al. [2001]). The chemical properties of these polymers allow hydrolytic degradation through de-esterification. Once degraded, the lactic and glycolic acid monomers are metabolized naturally by tissues. Due to these properties, PLA, PGA, PCL, and their copolymers have successfully been applied in a number of biomedical devices, such as degradable sutures and bone internal fixation devices (Biofix®, Bionx Implants Ltd., Tampere, Finland) which have been approved by the US Food and Drug administration (Mano et al. [2004]). However, abrupt release of these acidic degradation products can cause a strong inflammatory response (Bergsma et al. [1993]; Martin et al. [1996]). In general, their degradation rates decrease in the following order: PGA > PLA > PCL. Their blends have been shown to degrade faster than their pure counterparts (Dunn et al. [2001]). Poly lactate-glycolic acid (PLGA) can completely degrade in several months *in vivo*, whereas poly-L-lactate (PLLA) and PCL take 3 to 5 years or more to completely degrade *in vivo* (Rich et al. [2002]; Yang et al. [2001]).

Of particular significance for applications in tissue engineering is the acidic degradation products of PLA, PGA, PCL, and their copolymers that have been implicated in adverse tissue reactions (Niiranen et al. [2004]; Yang et al. [2001]). Researchers have incorporated basic compounds to stabilize the pH of the environment surrounding the polymer and to control its degradation, such as bioactive glasses and calcium phosphates (Dunn et al. [2001]; Heidemann et al. [2001]; Rich et al. [2002]). The possibility of counteracting this acidic degradation is another important reason proposed for the use of composites (Boccaccini and Maquet [2003]).

Other properties of thermoplastics of special interest include their excellent processability to generate a wide range of degradation rates, mechanical, and chemical properties achieved by the use of various molecular weights and stoichiometric ratios. Scaffolds produced in this can be mechanically strong and matched to specific tissue types, but their compliance is not reversible. Given that elastic stretchability is a major mechanical property of living tissue, including collagens of different bone types, elastomeric polymers that can provide sustainable elasticity and structural integrity are thought to be mechanically more advantageous than thermoplastic (non-elastomeric) polymers. Over the past 10 years, there have been an increasing number of research groups working on the development of biodegradable elastomeric biomaterials for bone tissue engineering applications (Li et al 2012; Kim and Mooney [2000]; Niklason et al. [1999]; Seliktar et al. [2003]; Stegemann and Nerem [2003]; Waldman et al. [2004]; Wang et al. [2002a]).

There is a family of hydrophobic polymers that undergo a heterogeneous hydrolysis process that is predominantly confined to the polymer-water interface. This property is referred to as surface eroding as opposed to bulk-degrading behavior. Three representative surface erodible polymers are poly(anhydrides) (poly(1,3-bis-p-carboxyphenoxypropane anhydride) (Domb and Langer [1999a]) and poly (erucic acid dimer anhydride) (Domb and Langer [1999b]), poly(ortho esters) (POE) (Andriano et al. [2002]; Solheim et al. [2000]), and polyphosphazenes (Allcock [2002]; Magill [1999]; (Laurencin et al. [1993, 1996b]). These surface bioeroding polymers have been intensively investigated as drug delivery vehicles. The surface-eroding characteristics offers three key advantages over bulk degradation when used as scaffold materials: (1) retention of mechanical integrity over the degrading lifetime of the device, owing to the maintenance of mass to volume ratio, (2) minimal toxic effects (i.e., local acidity), owing to lower solubility and concentration of degradation products, and (3) significantly enhanced bone ingrowth into the porous scaffolds, owing to the increment in pore size as the erosion proceeds (Shastri et al. [2002]).

## Biodegradable thermoplastic rubbers

Synthetic elastomers can be divided into two categories: thermoplastic elastomers and cross-linked elastomers, based on the type of ‘cross-link’ used to join their molecular chains. Unlike cross-linked elastomers, where the cross-link is a covalent bond created during the vulcanization process, the cross-link in thermoplastic elastomers is a weaker dipole or hydrogen bond,or takes place in one of the phases of the material. Linear thermoplastic elastomers usually consist of two separated microphases: crystalline, hydrogen-bonded hard segments and amorphous soft segments. The crystalline or hard segments function as cross-linkers which provide mechanical strength and stiffness, whereas soft segments provide the flexibility (Hiki et al. [2000]).

### Poly (ϵ-caprolactone) copolymers with glycolide or lactide

PCL, PGA, and PLA are rigid and have a poor flexibility. In order to provide better control over the degradation and mechanical properties without sacrificing biocompatibility, PCL-based materials have been copolymerized or blended with other hydroxyacids or polymers to produce elastomeric biomaterials. PCL-based copolymers with glycolide and lactide are elastomeric materials. Poly (lactide-co-caprolactone) (PLACL) synthesized by Cohn and Salomon ([2005]) demonstrates remarkable mechanical properties, with Young's modulus, UTS, and strain at break being up to 30 MPa, 32 MPa, and 600%, respectively.

The degradation rate of the PCL-based copolymers varies over a wide range by the change in the ratio of monomers. In general, the copolymers degrade faster than each homopolymer alone. PCL-co-GA scaffolds synthesized by Lee et al. ([2003]), for example, lost 3% of their initial mass after 2-week incubation in PBS and 50% after a 6-week incubation, whereas it takes 6–12 months and 2–3 years for PGA and PCL to degrade, respectively (Cohn and Salomon [2005]). PGA-co-CL (PGACL) and PLA-co-CL (PLACL) polymers were initially developed for engineering smooth muscle-containing tissues (e.g., blood vessels and urinary bladder) (Keun Kwon et al. [2005]; Lee et al. [2003]; Matsumura et al. [2003a, b]). Both were soon after investigated for their potential applications in bone tissue engineering (Gupta et al. [2009]; Webb et al. [2004]; Zilberman et al. [2005]).

### Polyhydroxyalkanoates

Polyhydroxyalkanoates are aliphatic polyesters as well, but produced by microorganisms under unbalanced growth conditions (Doi et al. [1995]; Li et al. [2005]). These polyesters are generally biodegradable (via hydrolysis) and thermoprocessable, making them attractive as biomaterials for medical devices and tissue engineering scaffolds (Chen and Wu [2005]). Polyhydroxybutyrate has been investigated for the repair of bone, nerves, blood vessels, urinary tissue, and those of the gastrointestinal tract.

Poly 3-hydroxybutarate (P3HB) is rigid and brittle, with a strain at break typically less than 5%. This thermoplastic material can easily be woven or compressed into textiles with a satisfactory flexibility (Chen and Wu [2005]). P3HB has been intensively investigated for bone tissue applications and produces a consistently favorable bone tissue adaptation response with no evidence of an undesirable chronic inflammatory response after implantation periods up to 12 months (Duvernoy et al. [1995]; Kalangos and Faidutti [1996]). Bone is formed close to the material and subsequently becomes highly organized, with up to 80% of the implant surface lying in direct apposition to newly mineralized bone. The materials showed no evidence of extensive structural breakdown *in vivo* during the implantation period of the study (Doyle et al. [1991]).

Among the PHAs, poly 4-hydroxybutyrate (Freier [2006]; Grabow et al. [2004]; Martin and Williams [2003]; Martin et al. [1999]; Rao et al. [2010]) and copolymers of 3-hydroxybutyrate and 4-hydroxybutyrate (Freier [2006]; Grabow et al. [2004]; Sudesh and doi [2005]), including P3HB-co-3HV (3-hydroxyvalerate) (Avella et al. [2000]), P3HB-co-3HD (3-hydroxydecanoate) (Avella et al. [2000]), and P3HB-co-3HH (3-hydroxyhexanoate), have been demonstrated to have superb elasticity, with an elongation at break of 400 to 1,100%. The major progress for these materials has so far been in cardiovascular tissue engineering (Martin and Williams [2003]; Shum-Tim et al. [1999]); however, for bone tissue engineering, P3HB-3HH showed improved attachment, proliferation, and differentiation of rabbit bone marrow cells (Wang et al. [2004]; Yang et al. [2004]) and chondrocytes (Deng et al. [2002, 2003]; Zhao et al. [2003a, b]; Zheng et al. [2003, 2005]) compared to PLLA. Despite the relatively small amount of research on their applications in bone and cartilage engineering, the potential of the above-mentioned soft elastomeric PHAs should not be ignored, and much research is needed to explore their application as bone engineering scaffolds.

### Polyurethane

Polyurethanes (PUs) are a large family of polymeric materials with an enormous diversity of chemical compositions, mechanical properties, tissue-specific biocompatibility, and biodegradability (Lamba et al. [1998]; Santerre et al. [2005]; Zdrahala [1996]). PUs are generally synthesized with three components: a diisocyanate, a polyol, and a chain extender (usually a diamine or diol) by step growth polymerization (Ganta et al. [2003]; Szycher [1999]). The resultant polyurethanes are phase-segregated polymers composed of alternating polydispersed blocks of ‘soft’ segments (made of macropolyols) and ‘hard’ segments (made of diisocyanates and chain extenders). Because of the differences in polarity between the hard (polar) and soft (nonpolar) segments, segmented PU elastomers can undergo microphase separation to form hard and soft domains. The soft domains are rubbery and amorphous at room temperature due to a glass transition temperature of less than 0°C. The hard domains, which result in the induction of hydrogen bonding between urethane and urea groups in the hard segments of adjacent polymer chains, function as physical cross-links that resist flow when stress is applied to the materials (Guelcher [2008]). The mechanical properties, as well as the biodegradation rate, can be tuned by modifying the structure of the hard and soft segments and/or changing the relative fractions of the hard and soft segments.

Historically, PUs had been used in permanent medical devices; they were actually subjected to hydrolysis, oxidation, and enzymatic degradation (Jayabalan et al. [2000]; Pinchuk [1994]). The soft segments generally dominated the degradation characteristics of PUs, and a high content of soft segments tends to increase the degradation rate (Pinchuk [1994]). Many attempts were made to resist biodegradation processes (Zdrahala [1996]). Converse to this, more recent attempts have been made to enhance the biodegradability of PUs. Over the past two decades, scientists have been utilizing the flexible chemistry of PU materials to design degradable polymers for tissue engineering, including both hard (Saad et al. [1997]) and soft types (Alperin et al. [2005]; Borkenhagen et al. [1998]; Fujimoto et al. [2007]; McDevitt et al. [2003]). These materials have taken advantage of processes such as hydrolytic mechanisms and have varied molecular structure to control hydrolysis rates.

In contrast to degradation behavior of PLA, PGA, and PLGA, PUs demonstrated no significant pH change in the microenvironment of their degradation products, instead showing a linear degradation rate with no autocatalytic effect (Guan et al. [2005]). However, the degradation products of PUs could be toxic when aromatic diisocyanates (e.g. 4,4′-methylenediphenyl diisocyanate and toluene diisocyanate) are used. To address this problem, aliphatic diisocyanates (e.g., lysine diisocyanate (LDI) and 1,4-diisocyanatobutane (BDI)) have been used as the replacements of aromatic diisocyanates (Gunatillake et al. [2003b]; Lamba et al. [1998]; Pinchuk [1994]) in PUs that are designed to be biodegradable.

In general, PUs are recognized to have good blood and tissue compatibility (Fromstein and Woodhouse [2006]; Zdrahala and Zdrahala [1999]). PUs made with LDI as the diisocyanate demonstrated no significantly detrimental effects on cell viability, growth, and proliferation *in vitro* and *in vivo*. Subcutaneous implantation in rats revealed that LDI-based PUs did not aggravate capsule formation, accumulation of macrophages, or tissue necrosis (Zhang et al. [2002]). Excellent reviews on biocompatibility of PUs can be found in a number of books (Fromstein and Woodhouse [2006]; Lamba et al. [1998]; Zdrahala and Zdrahala [1999]) and a number of topic reviews (Christenson et al. [2007]; Griesser [1991]; Guelcher [2008]; Santerre et al. [2005]; Szycher et al. [1996]; Zdrahala [1996]; Zdrahala and Zdrahala [1999]).

Most aliphatic diisocynate-based poly(ester urethane urea)s (PEUU)s have a Young's modulus (at small strains) of several tens of megapascals and an impressively large breaking strain in the range of 100 to 1,000% (Guan and Wagner [2005]; Hong et al. [2010]). PU rubbers made from PEUU: BDI/PCL, PEUU: BDI/PCL-polycarbonate, and PCUU: BDI/polycarbonate show a super elasticity, with the elongation at break and resilience being 600% to 800% and 99% to 100%, respectively, (Guan and Wagner [2005]; Hong et al. [2010]).

In addition to their tunable mechanical and biodegradable properties, PU elastomers also have a good processibility. They can be fabricated into highly porous scaffolds by a number of foaming techniques, such as thermally induced phase separation (Guan et al. [2005]) salt leaching/freeze-drying (Gogolewski and Gorna [2007]; Gogolewski et al. [2006]; Spaans et al. [1998a, 1998b]), wet spinning (Gisselfalt et al. [2002]; Liljensten et al. [2002]), and electrospinning (Stankus et al. [2004, 2007]). By applying the fabrication techniques mentioned above, different porosities, surface-to-volume ratios, and three-dimensional structures with concomitant changes in mechanical properties can be achieved to suit a wide range of tissue engineering, including bone and soft tissues (Guelcher [2008]). Table 1 provides a summary of the applications of PUs in bone tissue engineering.

**Table 1:** **Bone tissue engineering applications of polyurethanes**

Animal models	Polyurethane scaffolds	Major conclusions	Reference
Iliac crest (sheep)	Porous scaffolds synthesized from HMDI, PEO-PPO-PEO, and PCL at various ratios. Pore size, 300 to 2,000 μm; porosity, 85%	At 18 and 25 months, all the defects in the ilium implanted with polyurethane bone substitutes had healed with new bone.	Gogolewski and Gorna ([2007]), Gogolewski et al. ([2006])
		The extent of bone healing depended on the chemical composition of the polymer from which the implant was made.	
		The implants from polymers with the incorporated calcium-complexing additive were the most effective promoters of bone healing, followed by those with vitamin D and polysaccharide-containing polymer.	
		There was no bone healing in the control defects.	
Bone marrow stromal cells	BDI with PCL films	Bone marrow stromal cells were cultured on rigid polymer films under osteogenic conditions for up to 21 days. This study demonstrated the suitability of this family of PEUUs for bone tissue engineering applications.	Kavlock et al. ([2007])
Femoral condyle	LTI with PCL-co-PGA-co-PDLLA	Extensive cellular infiltration deep to the implant and new bone formation at 6 weeks	Dumas et al. ([2010])
Chondrocytes	Porous scaffolds synthesized from HMDI with PCL and ISO	Although the covalent incorporation of the isoprenoid molecule into the polyurethane chain modified the surface chemistry of the polymer, it did not affect the viability of attached chondrocytes.	Eglin et al. ([2010])
		The change of surface characteristics and the more open pore structure of the scaffolds produced from the isoprenoid-modified polyurethane are beneficial for the seeding efficiency and the homogeneity of the tissue-engineered constructs.	

From the point of view of biodegradation, PHAs and PUs could, in principle, be used in tissue engineering as implants that require a longer retention time or a higher stability in the surrounding environment, but which eventually absorb. This might be useful for tissues with slower healing and remodeling times or with an inability to maintain innate structural integrity (e.g., muscle). Their slow degradation profile (2 to 3 years) has limited their applications in bone tissue engineering, as the healing rate of bone is typically 6 to 12 weeks (Kakar and Einhorn [2008]). Hence, suitable elastomeric polymers with faster degradation kinetics that matches the healing profile of bone tissue remain to be explored. For this, recently developed degradable, chemically cross-linked polyester elastomers provide considerable potential (see the ‘Biodegradable chemically cross-linked elastomers’ section).

## Biodegradable chemically cross-linked elastomers

### Poly(propylene fumarate)

Poly(propylene fumarate) (PPF) is an unsaturated linear polyester. Like PLA and PGA, the degradation products of PPF (i.e., propylene glycol and fumaric acid) are biocompatible and readily removed from the body. The double bond along the backbone of the polymer permits cross-linking *in situ*, which causes a moldable composite to harden within 10 to 15 min. Mechanical properties and degradation time of the composite may be controlled by varying the PPF molecular weight. Therefore, preservation of the double bonds and control of molecular weight during PPF synthesis are critical issues (Payne and Mikos [2002]). PPF has been suggested for use as a scaffold for guided tissue regeneration, often as part of an injectable bone replacement composite (Yaszemski et al. [1995]). It also has been used as a substrate for osteoblast cultures (Peter et al. [2000]). The development of composite materials combining PPF and inorganic particles, e.g., HA or bioactive glass, has not been investigated to a large extent in comparison with the extensive research efforts dedicated to PLGA- and PLA-based composites.

### Poly(polyol sebacate)

Poly(polyol sebacate) (PPS) is a family of cross-linked polyester elastomers, developed for soft tissue engineering (Wang et al. [2002a]). Polyol and sebacic acid are both endogenous monomers found in human metabolites (Ellwood [1995]; Natah et al. [1997]; Sestoft [1985]); hence, PPSs generally show little toxicity to host tissues (Chen et al. [2011a]; Wang et al. [2003]). Poly(glycerol sebacate) (PGS) is the most extensively evaluated member of the PPS family, with most *in vitro* data demonstrating that PGS has a very good biocompatibility (Fidkowski et al. [2005a]; Gao et al. [2007]; Motlagh et al. [2006]; Sundback et al. [2005]; Sundback et al. [2004]; Wang [2004]). Poly(xylitol sebacate) (PXS) has also been developed using xylitol, a well-studied monomer in terms of biocompatibility and pharmacokinetics in humans (Ellwood [1995]; Natah et al. [1997]; Sestoft [1985]; Talke and Maier [1973]). As a metabolic intermediate in the mammalian carbohydrate metabolism, xylitol enters the metabolic pathway slowly without causing rapid fluctuations of blood glucose levels (Natah et al. [1997]; Winkelhausen and Kuzmanova [1998]). Inspired by the good biocompatibility of xylitol, Langer's group was the first to develop PXS (Bruggeman et al. [2008b, 2010]). An *in vitro* evaluation of biocompatibility of PXS, poly(sorbitol sebacate) (PSS), and poly(mannitol sebacate) (PMS) polymers showed that they supported primary human foreskin fibroblasts in terms of cellular attachment and proliferation with the exception of PSS and PMS that were synthesised at the ratio of 1:1 (polyol/sebacic acid) (Bruggeman et al. [2008a]).

*In vivo* assessment of PGS was first conducted by subcutaneous implantation of 3-mm-thick material in Sprague–Dawley rats (Wang et al. [2002b, 2003]; Wang [2004]). This evaluation showed that PGS induced an acute inflammatory response but no chronic inflammation, while PLGA caused both. The PGS implants in rats were completely absorbed after 60 days without scarring or permanent damage to tissue structure (Wang [2004]). Another *in vivo* investigation via subcutaneously implanted PGS films in the same species has shown that PGS has excellent biocompatibility, inducing only a mild inflammatory response (Pomerantseva et al. [2009]). *In vivo* applications of PGS in the nerve (Sundback et al. [2005]), vascular (Bettinger et al. [2005b, 2006]; Kemppainen and Hollister [2010]; Motlagh et al. [2006]), and myocardial (Stuckey et al. [2010]) tissue engineering consistently show a mild foreign body response in terms of both acute and chronic inflammations. Subcutaneous implantation of PXSs in Lewis rats has shown improved biocompatibility when compared to PLGA implants (Bruggeman et al. [2008b]). Up to now, reports on PXS have indicated that these elastomers could be viable candidates as biodegradable medical devices that can offer structural integrity and stability over a clinically required period (Bruggeman et al. [2010]). PSS and PMS polymers also exhibit better *in vivo* biocompatibility than PLGA, evidenced by mild acute inflammatory reactions and less fibrous capsules formation during chronic inflammation (Bruggeman et al. [2008a]).

PGS was reported to be completely resorbed 60 days after implantation in rats (Wang et al. [2003]). This comparatively faster degradation rate of PGS *in vivo* was also reported by Stuckey et al. ([2010]) who used PGS sheets as a pericardial heart patch. They found that the PGS patch was completely resorbed after 6 weeks. These examples of *in vivo* degradation indicate that aqueous enzymatic action, combined with dynamic tissue movements and vascular perfusion, might enhance the enzymatic breakdown of ester bonds in PGS and, thus, facilitate the hydrolytic weakening of this material *in vivo*.

Most recently, an *in vitro* enzymatic degradation protocol was reported to be able to simulate and quantitatively capture the features of *in vivo* degradation of PGS-based materials (Liang et al. [2011]). In the study, PGS and PGS/Bioglass® composites were subjected to enzymatic degradation in tissue culture medium or a buffer solution at the pH optima in the presence of defined concentrations of an esterase. The *in vitro* enzymatic degradation rates of the PGS-based materials were markedly higher in the tissue culture medium than in the buffered solution at the optimum pH 8. The *in vitro* enzymatic degradation rate of PGS-based biomaterials cross-linked at 125°C for 2 days was approximately 0.5 to 0.8 mm/month in tissue culture medium, which falls within the range of *in vivo* degradation rates (0.2 to 1.5 mm/month) of PGS cross-linked at similar conditions. Enzymatic degradation was also further enhanced in relation to cyclic mechanical deformation.

Briefly, PGS and the related PPS family are rapidly degrading polymers (several weeks) (Chen et al. [2012b]; Li et al. [2012]; Liang et al. [2011]). Up to now, there is only one report on the application of PPS as a scaffolding material for bone tissue engineering (Chen et al. [2010d]). Nonetheless, it must be emphasized that *among the above-reviewed degradable polymers, the rapid degradation kinetics of the PPS family best matches the healing profile of bone, which has complete healing rates of 6 to 12 weeks* (Kakar and Einhorn [2008]).

## Bioactive ceramics

A common feature of bioactive glasses and ceramics is a time-dependent, kinetic modification of the surface that occurs upon implantation. The surface forms a biologically active hydroxycarbonate apatite (HCA) layer, which provides the bonding interface with tissues. The HCA phase that forms on bioactive implants is chemically and structurally equivalent to the mineral phase in bone, providing interfacial bonding (Hench [1991, 1998]). The *in vivo* formation of an apatite layer on the surface of a bioactive ceramic can be reproduced in a protein-free and acellular simulated body fluid, which is prepared to have an ionic composition similar to that of the human blood plasma, as described previously (Kokubo et al. [2003]). Typical mechanical properties of the bioactive ceramic phases discussed in this article are listed in Table 2.

**Table 2:** **Mechanical properties of hydroxyapatite, 45 S5 Bioglass®, glass-ceramics, and human cortical bone**

Ceramics	Compression strength (MPa)	Tensile strength (MPa)	Elastic modulus (GPa)	Fracture toughness$\text{(MPa}\sqrt{\text{m)}}$	Reference
Hydroxyapatite	>400	approximately 40	approximately 100	approximately 1.0	Hench ([1999]), LeGeros and LeGeros ([1999])
45 S5 Bioglass®	approximately 500	42	35	0.5 to 1	Hench ([1999]), Hench and Kokubo ([1998])
A-W	1,080	215 (bend)	118	2.0	Kokubo ([1999b])
Parent glass of A-W	NA	72 (bend)	NA	0.8	Kokubo ([1999b])
Bioverit® I	500	140 to 180 (bend)	70 to 90	1.2 to 2.1	Holand and Vogel ([1993])
Cortical bone	130 to 180	50 to 151	12 to 18	6 to 8	Keaveny and Hayes ([1993]), Moore et al. ([2001]), Nalla et al. ([2003]), Zioupos and Currey ([1998])

NA, not applicable.

### Dilemmas in developing biomaterials for bone tissue engineering

Since almost two-thirds of the weight of bone is hydroxyapatite Ca_10_(PO_4_)_6_(OH)_2_, it seems logical to use this ceramic as the major component of scaffold materials for bone tissue engineering. Actually, hydroxyapatite and related calcium phosphates (CaP) (e.g., β-tricalcium phosphate) have been intensively investigated ([1990]; Burg et al. [2000]; Hench and Wilson [1999]; LeGeros and LeGeros [2002]). As expected, calcium phosphates have an excellent biocompatibility due to their close resemblance to bone mineral chemical and crystal structure (Jarcho [1981]; Jarcho et al. [1977]). Although they have not shown osteoinductive ability, they certainly possess osteoconductive properties as well as a remarkable ability to bind directly to bone (Denissen et al. [1980]; Driskell et al. [1973]; Hammerle et al. [1997]; Hollinger and Battistone [1986]). A large body of *in vivo* and *in vitro* studies have reported that calcium phosphates, no matter in which form (bulk, coating, powder, or porous) or phase (crystalline or amorphous) they are in, consistently support the attachment, differentiation, and proliferation of osteoblasts and mesenchymal cells, with hydroxyapatite being the best one among them (Brown et al. [2001]).

Crystalline calcium phosphates have long been known to have very prolonged degradation times *in vivo*, often in the order of years (Rezwan et al. [2006]; Vacanti et al. [2000]). Nanosized carbonated HA is a stable component of natural bone, though it metabolizes like all tissues. Hence, it would be fundamentally wrong if one expected HA to rapidly degrade in a physiological environment. In fact, clinical investigation has recently demonstrated *that implanted hydroxyapatites and calcium phosphates are virtually inert*, remaining within the body for as long as 6 to 7 years post-implantation (Marcacci et al. [2007]). This should make HA less favored as a scaffold material for use in tissue engineering. The degradation rates of amorphous HA and TCP are high, but they are too fragile to build a 3D porous network.

The properties of synthetic calcium phosphates vary significantly with their crystallinity, grain size, porosity, and composition. In general, the mechanical properties of synthetic calcium phosphates decrease significantly with increasing content of amorphous phase, microporosity, and grain size. High crystallinity, low porosity, and small grain size tend to give higher stiffness, higher compressive and tensile strength, and greater fracture toughness (Kokubo [1999a]; LeGeros and LeGeros [1999]). It has been reported that the flexural strength and fracture toughness of dense hydroxyapatite are much lower in dry compared to aqueous conditions (de Groot et al. [1990]).

Comparing the properties of hydroxyapatite and related calcium phosphates with those of bone (Table 2), it is apparent that the bone has a reasonably good compressive strength, though it is lower than that of hydroxyapatite, and better tensile strength and significantly better fracture toughness than hydroxyapatite. The apatite crystals in the bone tissue make it strong enough to tolerate compressive loading. Combined with macroscale stress fibers, and the typically tubular structure of long bone or mesh-like structure of flatter bone, the high tensile strength and fracture toughness are attributed to flexible collagen fibers. Hence, calcium phosphates alone cannot be used for load-bearing scaffolds in spite of their good biocompatibility and osteoconductivity.

A major challenge in bone tissue engineering is to develop a scaffolding material that is mechanically strong and yet biodegradable. To engineer bone tissue, which is hard and functions to support the body, the scaffold material must be strong and tough. Ideally, the scaffold needs to be degradable, as this biodegradation would avoid the detrimental effects of a persisting foreign substance and allow its gradual replacement with the new bone. Unfortunately, in this context, *mechanical strength and biodegradability counteract each other*. In general, mechanically strong materials (e.g., crystalline hydroxyapatite, Ti alloys, and crystalline polymers) are virtually inert and remain part of the repaired bone, while biodegradable materials (e.g., amorphous hydroxyapatite and glasses) tend to be mechanically fragile. This forms the greatest challenge in the design of bioceramics for bone engineering at load-bearing sites, but there are processing approaches such as sintering of 45 S5 Bioglass® (Chen and Boccaccini [2006a]), for example, may offer opportunities to address the above dilemma (see the ‘Na-containing silicate bioactive glasses’ section).

### Na-containing silicate bioactive glasses

The basic constituents of the most bioactive glasses are SiO_2_, Na_2_O, CaO, and P_2_O_5_. 45 S5 Bioglass® contains 45% SiO_2_, 24.5% Na_2_O, 24.4% CaO, and 6% P_2_O_5_, in weight percent (Hench [1991]). In 1969, Hench and co-workers discovered that certain glass compositions had excellent biocompatibility as well as the ability to bond bone (Hench et al. [1971]). The bioactivity of this glass system can vary from surface bioactive (i.e., bone bonding) to bulk degradable (i.e., resorbed within 10 to 30 days in tissue) (Hench [1998]). Through interfacial and cell-mediated reactions, bioactive glass develops a calcium-deficient, carbonated phosphate surface layer that allows it to chemically bond to host bone (Hench [1997–1999]; Hench et al. [1971]; Hench and Wilson [1993]; Pereira et al. [1994]; Wilson et al. [1981]). It is clearly recognized that for a bond with bone tissue to occur, a layer of biologically active carbonated hydroxyapatite (HCA) must form (Hench and Wilson [1984]). This bioactivity is not exclusive to bioactive glasses; hydroxyapatite and related calcium phosphates also show an excellent ability to bond to bone, as discussed further below. The capability of a material to form a secure biological interface with the surrounding tissue is critical in the elimination of scaffold loosening.

An important feature of bioactive glasses for applications in bone tissue engineering is their ability to induce bone tissue growth processes such as enzyme activity (Aksay and Weiner [1998]; Lobel and Hench [1996, 1998]; Ohgushi et al. [1996]), revascularization (Day et al. [2004]; Keshaw et al. [2005]), osteoblast adhesion and differentiation from mesenchymal stem cells (Gatti et al. [1994]; Lu et al. [2005]; Roether et al. [2002]; Schepers et al. [1991]). Another significant finding is that the dissolution products from bioactive glasses, in particular the 45 S5 Bioglass® composition, upregulate osteogenic gene expression and growth factor production (Xynos et al. [2000a]). Silicon alone has been found to play a key role in bone mineralization and gene activation, which has led to an increased interest in the substitution of silicon for calcium into synthetic hydroxyapatites. Investigations *in vivo* have shown that bone ingrowth into silicon-substituted HA granules was remarkably greater than that into pure HA (Xynos et al. [2000b]).

It has been found that bioactive glass surfaces can release biologically relevant levels of soluble ionic forms of Si, Ca, P, and Na, depending on the processing route and particle size. These released ions induce intracellular and extracellular responses (Xynos et al. [2000a, 2001]). For example, a synchronized sequence of genes is activated in the osteoblasts that undergo cell division and synthesize an extracellular matrix, which mineralizes to become bone (Xynos et al. [2000a, 2001]). In addition, bioactive glass compositions doped with AgO_2_ have been shown to elicit antibacterial properties while maintaining their bioactive function (Bellantone et al. [2002]). In recent investigations, 45 S5 Bioglass® has been shown to increase secretion of vascular endothelial growth factor *in vitro* and to enhance vascularization *in vivo*, suggesting that scaffolds containing controlled concentrations of Bioglass® might stimulate neovascularization, which is beneficial to large tissue constructs (Day et al. [2004]).

One key reason that makes bioactive glasses a relevant scaffold material is the possibility of controlling a range of chemical properties and, thus, the rate of bioresorption. The structure and chemistry of glasses, in particular sol–gel derived glasses (Pereira et al. [1994]); Chen et al. [2010b]; Chen and Thouas [2011]), can be tailored at a molecular level by modifying the thermal or environmental processing history to vary the composition. It is possible to design glasses with degradation properties specific to a particular application of bone tissue engineering.

It was once reported that crystallization of bioactive glasses, which is necessary to achieve mechanical strength, decreased the level of bioactivity (Filho et al. [1996]), even turning a bioactive glass into an inert material (Li et al. [1992]). This antagonism between bioactivity and mechanical strength was considered to hamper the application of bioactive glasses. This issue has now been addressed by the discovery that Na-containing glasses (e.g., 45 S5 Bioglass®) can be sintered to form a mechanically strong crystalline phase, which can transform into amorphous calcium phosphate at body temperature and in a biological environment, remaining both bioactive and degradable (Chen and Boccaccini [2006a]; Chen and Boccaccini [2006b]; Chen et al. [2011c, 2012a]; Chen [2011]). The loss in mechanical strength due to biodegradation is in the time fashion of tissue engineering, i.e., matching the healing profile of bone. This highly desirable property is a unique feature of this 45 S5 Bioglass®, which has not previously been found in any other material (e.g., hydroxyapatites, Ti-alloys, or polymers).

The above advantages are the reasons why 45 S5 Bioglass® is relatively successfully exploited in clinical treatments of periodontal disease (Perioglas^TM^) and as a bone filler material (Novabone^TM^) (Hench [1998]). Bioglass® implants have also been used to replace damaged middle ear bones, restoring hearing to patients (Hench [1997]). Bioactive glasses have gained new attention recently as promising scaffold materials, either as fillers or coatings of polymer structures, and as porous materials themselves when melt-derived and sol–gel-derived glasses (Boccaccini and Maquet [2003]; Boccaccini et al. [2003]; Chen and Boccaccini [2006b]; Chen et al. [2010c]; Chen and Thouas [2011]; Jones and Hench [2003a, b]; Kaufmann et al. [2000]; Laurencin et al. [2002]; Livingston et al. [2002]; Yuan et al. [2001]).

### Borate bioactive glasses

While silicate 45 S5 compositions have been widely investigated over the last 50 years, borate- and borosilicate-based compositions have recently been explored (Fu et al. [2012]; Rahaman et al. [2011]; Yang et al. [2012]). Boron is a trace element (see the ‘Bioactive glasses doped with trace elements’ section). Dietary boron is documented to benefit in bone health (Nielsen [2008]; Uysal et al. [2009]), as shown by Chapin et al. ([1997]). In their study, rats developed improved vertebral resistance to crash force after dietary intake of boron (Chapin et al. [1997]). Gorustovich et al. ([2006, 2008]) furthermore found that boron deficiency in mice alters periodontal alveolar bone remodeling by inhibiting bone formation.

Borate bioactive glasses have been reported to support cell proliferation and differentiation *in vitro* (Fu et al. [2009], 2010a; Marion et al. [2005]) and tissue infiltration *in vivo* (Fu et al. [2010b]). Boron concentrations in the blood around borate glass pellets implantation in rabbit tibiae were well below the toxic level (Zhang et al. [2010]). However, there is a concern associated with the toxicity of boron released into the solution as borate ions, (BO_3_)^3−^. It has been reported that some borate glasses exhibited cytotoxicity under static *in vitro* culture conditions (Fu et al. [2010b]), although no considerable toxicity was detected under more dynamic culture conditions, suggesting the importance of borate clearance (Fu et al. [2010b]).

Borate bioactive glasses have also been reported to degrade faster than their silicate counterparts due to their relative chemical instability (Fu et al. [2009, 2010a], c; Huang et al. [2006a, 2007]; Yao et al. [2007]). By partially or fully replacing the SiO_2_ in silicate glasses with B_2_O_3_, the complete degradation rate of the glasses can be varied over a wide range, from several days to longer than 2 months (Fu et al. [2009, 2010a], c; Huang et al. [2006a]; Yao et al. [2007]). Moreover, borate bioactive glasses are more readily converted to an apatite-like composition than the silicate materials (Huang et al. [2006a]). The conversion mechanism of bioactive glass to apatite is similar to that of silicate 45 S5 glass, with the formation of a borate-rich layer, similar to the silicate-rich layer of the former (Hench [1998]; Huang et al. [2006a, b]). The ease of controlling the degradation rate in these borate-based glasses offers new opportunities to regulate the degradation rate of synthetic biomaterials to match injured bone healing rates.

### Bioactive glasses doped with trace elements

Bioactive glasses have recently modified by doping with elements such as Cu, Zn, and Sr, which are known to be beneficial for healthy bone growth (Fu et al. [2010a]; Hoppe et al. [2011]; Wang et al. [2011]; Zheng et al. [2012]). To understand the biological significances of these types of trace elements in materials, it is useful to consider their abundance in biological tissues. The most abundant compound in the human body is water (65 to 90 wt.%), which contains most of the oxygen and hydrogen (Table 3)**.** Approximately 96% of the weight of the body is comprised of oxygen, carbon, hydrogen, and nitrogen, which are the building blocks of all proteins. The rest (approximately 4%) of the mass of the body exists largely either in the bone and tooth as minerals (Ca, Mg, and P) or in the blood and extracellular fluid as major electrolytes (Na, K, and Cl), referred to here as macroelements (Table 4, reference).

**Table 3:** **Elements in the human body (Seeley et al.**[2006]**)**

Element	O	C	H	N	Ca	P	K	S	Na	Cl	Mg	Trace element
Wt.%	65.0	18.5	9.5	3.3	1.5	1.0	0.4	0.3	0.2	0.2	0.1	<0.01
At.%	25.5	9.5	63.0	1.4	0.31	0.22	0.06	0.05	0.3	0.03	0.1	<0.01

**Table 4:** **Macroelements and their roles in the human body (Whitney and Rolfes**[2010]**)**

Macroelements	Roles
O, C, H, N	In water and the molecular structures of proteins
Ca	Structure of bone and teeth; muscle and nerve activity
P	Structure of bone and teeth; intermediate in REDOX metabolism and production of ATP in energy
Mg	Important in bone structure, muscle contraction, and metabolic processes
Na	Major electrolyte of blood and extracellular fluid; required for the maintenance of pH and osmotic balance; nerve and muscle signaling
K	Major electrolyte of blood and intracellular fluid; required for the maintenance of pH and osmotic balance; nerve and muscle signaling
Cl	Major electrolyte of blood and extracellular and intracellular fluid; required for the maintenance of pH and osmotic balance; nerve and muscle signaling
S	Element of the essential amino acids methionine and cysteine; contained in the vitamins thiamine and biotin. As part of glutathione, it is required for detoxification. Poor growth due to reduced protein synthesis and lower glutathione levels potentially increasing oxidative or xenobiotic damage are consequences of low sulfur and methionine and/or cysteine intake.

In addition to the macroelements, there are also a large number of elements in lower concentrations (how much…ppm?) for the proper growth, development, and physiology of the body (see the list of known trace elements in the human body (Whitney and Rolfes [2010]) below). These elements are referred to as trace elements or micronutrients, and while this list is increasing, it is important to bear in mind that these trace elements are all toxic at high levels. In 1966, for instance, the addition of cobalt compounds to stabilize beer foam in Canada led to cardiomyopathy, which came to be known as *beer drinker's cardiomyopathy* ([1967]; Barceloux [1999]). In brief, the majority of metal elements are needed in the human body as micronutrients (eg., as enzyme cofactors) but are toxic at levels higher than required, partly resulting in excretion or excess storage as deposits. Hence, it is highly important that as a glass degrades *in vivo*, the trace elements in scaffolds must be released at a biologically acceptable rate. In this section, we focus on trace elements doped in bioactive glasses for bone tissue engineering, including strontium, zinc, and copper.

List of known trace elements in the human body, which are all toxic at high levels (Whitney and Rolfes [2010]).

• Barium

• Beryllium

• Boron

• Caesium

• Chromium

• Cobalt

• Copper

• Iodine

• Iron

• Lithium

• Molybdenum

• Nickel

• Selenium

• Strontium

• Tungsten

• Zinc

Strontium is chemically closely related to calcium, sharing the same main group with calcium on the periodic table of elements and having a similar atomic radius to the calcium cation (*r*_Sr_ = 1.16 Å and *r*_Ca2+_ = 1.0 Å). Because of the above chemical analogy, Sr has long been used as a dope element in the hydroxyapatite products (Chen et al. [2004]; Marie et al. [2001]; Wong et al. [2004]). *In vivo* investigations have demonstrated that strontium is, in general, a benign element, having pharmacological effects on bone balance in normal bone and in the treatment of osteoporosis (Marie et al. [2001]; Marie [2010]; Meunier et al. [2002]). Moreover, a drug of strontium ranelate has been reported to enhance fracture healing of bone in rats in terms of callus resistance. The group treated with only strontium ranelate showed a significant increase in callus resistance compared to the untreated control group. An added benefit of doping trace elements is the enhanced X-ray imaging contrast.

Zinc is necessary in the function of all cells, binding specific DNA regions to regulate genetic control of cell proliferation (Whitney and Rolfes [2010]). Zn is also reported to play a role in bone healing and metabolism (Yamaguchi [1998]), with anti-inflammatory roles (Lang et al. [2007]). It has been demonstrated that Zn (a) stimulates bone formation *in vitro* by activating protein synthesis in osteoblast cells, (b) increases ATPase activity in bone (Yamaguchi [1998]) and inhibits bone resorption of osteoclast cells in mouse marrow cultures (Yamaguchi [1998]), and (c) has regulatory effects on bone cells and, thus, on gene expression (Cousins [1998]; Kwun et al. [2010]). Nonetheless, it has been well documented that an excess of zinc may cause anemia or reduced bone formation (Whitney and Rolfes [2010]) as well as systemic cytotoxicity.

Copper is contained in enzymes of the ferroxidase (ceruloplasmin) system which regulates iron transport and facilitates release from storage. A copper deficiency can result in anemia from reduced ferroxidase function. However, excess copper levels cause liver malfunction and are associated with the genetic disorder Wilson's disease. There have been controversial reports on the effects of copper on bone remodelling. On the one hand, Zhang et al. ([2003]) reported that Cu^2+^ at a concentration of 10^−6^ M inhibits osteoclast activity. Smith et al. ([2002]) also found that dietary copper deprivation causes a reduction of bone mineral density. On the other hand, Cashman et al. ([2001]) found that copper supplements over a period of 4 weeks did not affect bone formation or bone resorption, as manifested by biochemical markers. Furthermore, Lai and Yamaguchi ([2005]) showed that supplementation with copper induced a decrease in bone tissue in rats, showing reduced or absent anabolic effects on bone formation both *in vivo* and *in vitro*.

Perhaps what is positively relevant to bone tissue engineering about copper is that this element has consistently been reported to trigger endothelial cells towards angiogenesis. Finney et al. ([2009]) found that a significant amount of Cu ions was distributed in human endothelial cells when they were induced to enhance angiogenesis. This phenomenon was believed to indicate the importance of copper ions as angiogenic agent. In another work, copper and angiogenesis growth factor FGF-2 were found to have synergistic stimulatory effects on angiogenesis *in vitro* (Gerard et al. [2010]). In addition to its function of stimulating proliferation of human endothelial cells (Hu [1998]), Cu was shown to promote the differentiation of mesenchymal stem cells towards the osteogenic lineage (Rodriguez et al. [2002]).

In summary, although trace elements have beneficial effects on bone remodeling and/or associated angiogenesis, the risk of toxicity at high levels must be highly regarded in the design of composition and degradation rate of bioactive biomaterials so that the release of these elements must be satisfactorily lower than their biologically safe levels.

## Biocomposites

The primary disadvantage of bioactive glasses is their mechanical weakness and low fracture toughness (Table 2) due to their amorphous structure. Hence, bioactive glasses alone have limited application in load-bearing situations owing to poor mechanical strength and mismatch with the surrounding bone. However, these materials can be sintered to improve their mechanical properties (Chen et al. [2006]a, Chen et al. 2006b), or used in combination with polymers to form composite materials with better bone repair potential (Roether et al. [2002]).

### Thermoplastic-based composites

From a biological perspective, it is a natural strategy to combine polymers and ceramics to fabricate scaffolds for bone tissue engineering because, structurally, native bone is essentially the combination of a naturally occurring polymer and biological apatite. From the materials science point of view, a single material type does not always provide the necessary mechanical and/or chemical properties desired for this particular application. In these instances, composite materials designed to combine the advantages of both materials may be most appropriate. Polymers and ceramics that degrade *in vivo* should be chosen for designing biocomposites for tissue engineering scaffolds, except for permanent implants. While massive release of acidic degradation from polymers causes inflammatory reactions (Bergsma et al. [1993, 1995]; Temenoff et al. [2000]), the basic degradation of calcium phosphate or bioactive glasses would buffer these by-products of polymers thereby improving the physiological conditions of tissue environment due to pH control. Mechanically, bioceramics are much stronger than polymers and play a critical role in providing mechanical stability to construct prior to synthesis of new bone matrix by cells. However, as mentioned above, ceramics and glasses are very fragile due to their intrinsic brittleness and flaw sensitivity. To capitalize on their advantages and minimize their shortcomings, ceramic and glass materials can be combined with various polymers to form composite biomaterials for osseous regeneration. Tables 5 and 6 list selected dense and porous ceramic/glass-polymer composites, which have been designed as biomedical devices or scaffold materials for bone tissue engineering, and their mechanical properties.

**Table 5:** **Biocomposites used for bone tissue engineering**

Biocomposite		Percentage of ceramic (%)	Compressive (*** C ***), tensile (*** T ***), flexural (*** F ***), and bending (*** B ***) strengths (MPa)	Modulus (MPa)	Ultimate strain (%)	Toughness (kJ/m^2^)	Reference
Ceramic	Polymer						
HA fiber	PDLLA	2 to 10.5 (vol.)	45 (*F*)	1.75× 10^3^ to 2.47 × 10^3^			Deng et al. ([2001])
	PLLA	10 to 70 (wt.)	50 to 60 (*F*)	6.4 × 10^3^ to 12.8 × 10^3^	0.7 to 2.3		Kasuga et al. ([2001])
HA	PLGA	40 to 85 (vol.)	22 (*F*)	1.1 × 10^3^		5.29	Xu et al. ([2004]), Xu and Simon ([2004a, b])
	Chitosan	40 to 85 (vol.)	12 (*F*)	2.15 × 10^3^		0.092	Xu et al. ([2004])
	Chitosan + PLGA	40 to 85 (vol.)	43 (*F*)	2.6 × 10^3^		9.77	Xu et al. ([2004])
	PPhos	85 to 95 (wt.)					Greish et al. ([2005])
	Collagen	50 to 72 (wt.)					Rodrigues et al. ([2003])
β-TCP	PLLA-co-PEH	75 (wt.)	51 (*F*)	5.18 × 10^3^			Kikuchi et al. ([1999])
	PPF	25 (wt.)	7.5 to 7.7 (*C*)	191 to 134			Peter et al. ([1998])
A/W	PE	10 to 50 (vol.)	18 to 28 (*B*)	0.9 × 10^3^ to 5.7 × 10^3^			Juhasz et al. ([2003a, b]), Juhasz et al. ([2004])
Ca_3_(CO_3_)_2_	PLLA	30 (wt.)	50	3.5 × 10^3^ to 6 × 10^3^			Kasuga et al. ([2003])
**Bioglass®**	**PGA**	**2 to 1 (wt.)**	**0.5 to 2 (** *** T *** **)**	**0.5 to 2 (** *** T *** **)**	**150 to 600**		Chen et al. ([2010a]) Chen et al. ([2011b]), Liang et al. ([2010])
Human cortical bone		70 (wt.)	50 to 150 (*T*)	12 × 10^3^ to 18 × 10^3^			Keaveny and Hayes ([1993]), Moore et al. ([2001]), Nalla et al. ([2003]), Zioupos and Currey ([1998])
			130 to 180 (*C*)

**Table 6:** **Properties of porous composites developed for bone tissue engineering**

Biocomposite		Percentage of ceramic (wt.%)	Porosity (%)	Pore size (μm)	Strength (MPa)	Modulus (MPa)	Ultimate strain (%)	Reference
Amorphous CaP	PLGA	28 to 75	75	>100		65		Ambrosio et al. ([2001]), Khan et al. ([2004])
β-TCP	Chitosa-gelatin	10 to 70		322 to 355	0.32 to 0.88	3.94 to 10.88		Yin et al. ([2003])
HA	PLLA	50	85 to 96	100 × 300	0.39	10 to 14		Zhang and Ma ([1999])
	PLGA	60 to 75	81 to 91	800 to 1800	0.07 to 0.22	2 to 7.5		Guan and Davies ([2004])
	PLGA		30 to 40	110 to 150		337 to 1459		Devin et al. ([1996])
Bioglass®	PLGA	75	43	89	0.42	51		Laurencin et al. ([2002]), Lu et al. ([2003]), Stamboulis et al. ([2002])
	PLLA	20 to 50	77 to 80	approximately 100 (macro); approximately 10 (micro)	1.5 to 3.9	137 to 260	1.1 to 13.7	Zhang et al. ([2004])
	PLGA	0.1 to 1		50 to 300				Blaker et al. ([2004])
	PDLLA	5 to 29	94	approximately 100 (macro); 10 to 50 (micro)	0.07 to 0.08	0.65 to 1.2	7.21 to 13.3	Blaker et al. ([2003, 2005]), Verrier et al. ([2004])
Phosphate glass A/W	PLA-PDLLA	40	93 to 97	98 to 154	0.017 to 0.020	0.075 to 0.12		Navarro, et al. ([2004]), Li and Chang ([2004])
	PDLLA	20 to 40	85.5 to 95.2					
**Bioglass**	**PGS**	**90**	**>90**	**300 to 500**	**0.4 to 1.0**			Chen et al. ([2010d])
Human cancellous bone		70	60 to 90	300 to 400	0.4 to 4.0	100 to 500	1.65 to 2.11	Giesen et al. ([2001]), Yeni and Fyhrie ([2001]), Yeni, et al. ([2001])

In general, all of these synthetic composites have good biocompatibility. Kikuchi et al. ([1999]), for instance, combined TCP with PLA to form a polymer-ceramic composite, which was found to possess the osteoconductivity of β-TCP and the degradability of PLA. The research team led by Laurencin synthesized similar porous scaffolds containing PLGA and HA, which combine the degradability of PLGA with the bioactivity of HA, fostering cell proliferation and differentiation as well as mineral formation (Attawia et al. [1995]; Devin et al. [1996]; Laurencin et al. [1996a]). Other composites of bioactive glass and PLA were observed to form calcium phosphate layers on their surfaces and support rapid and abundant growth of human osteoblasts and osteoblast-like cells when cultured *in vitro* (Blaker et al. [2004]; Blaker et al. [2003]; Blaker et al. [2005]; Boccaccini et al. [2003]; Li and Chang [2004]; Lu et al. [2003]; Maquet et al. [2003, 2004]; Navarro et al. [2004]; Stamboulis et al. [2002]; Verrier et al. [2004]; Zhang et al. [2004]).

A comparison between dense composites and cortical bone indicates that with thermoplastics, the most promising synthetic composite seems to be HA fiber-reinforced PLA composites (Kasuga et al. [2001]), which however exhibit mechanical property values closer to the lower values of the cortical bone. Up to now, the best thermoplastic-based composite scaffolds reported in the literature seem to be those made from combinations of Bioglass® and PLLA or PDLLA (Blaker et al. [2004]; Maquet et al. [2003, 2004]; Zhang et al. [2004]). These composites have a well-defined porous structure; at the same time, their mechanical properties are close to (but lower than) those of cancellous bone.

### Elastomer-based composites

**Figure 1:**
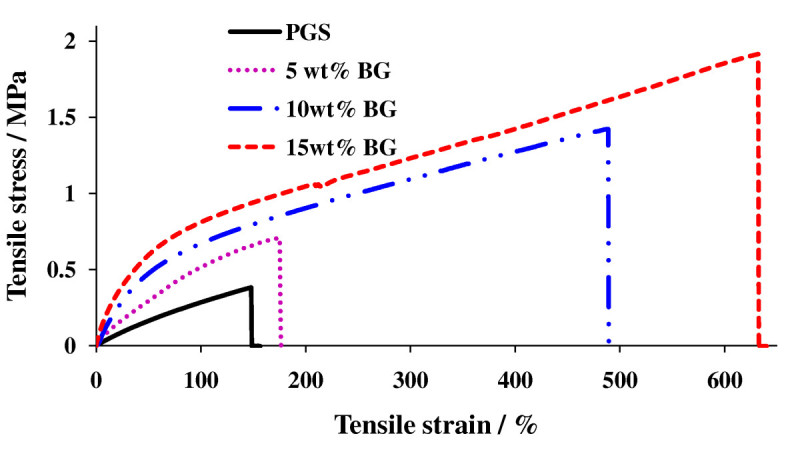
**Typical tensile stress–strain curves.** Of pure PGS and PGS composites of 5, 10, or 15 wt.% Bioglass®. Note the mechanical strength and strain at rupture increased simultaneously with the addition of Bioglass® filler (Chen et al. [2010a]; Liang et al. [2010]).

Very recently, our group developed elastomeric composites from PPS and bioceramics (Chen et al. [2010a, 2011b]; Liang et al. [2010]). There are several advantages of using PPS elastomers over other thermoplastic polymers as a base for a reinforced composite. Firstly, its elastomeric properties make it ideal for a range of tissue repair applications (Bettinger et al. [2005a]; Chen et al. [2008a]; Fidkowski et al. [2005b]; Redenti et al. [2009]; Wang et al. [2002b], c). In the case of bone, there is a requirement for some flexibility in the initial phases of bone repair, which involves cartilage deposition before bone formation (Oliveira et al. [2009]). Secondly, PPS is acidic and, thus, able to react with alkaline Bioglass® via metallic carboxylation, resulting in a chemical bonding between the inorganic and organic components of the composite (Ma and Wu [2007]). Thirdly, the degradation kinetics of PPS are entirely tunable by alternating its cross-link density to such a degree that it can maintain high physical integrity during degradation (Wang et al. [2002b]). In addition, the elastic properties (i.e., Young's modulus, elongation at break and resilience) of these composites can be enhanced simultaneously by adding ceramic fillers due to the bound-rubber mechanism (Figure 1) (Chen et al. [2010a, 2011b]; Liang et al. [2010]). Finally, due to its combination of satisfactory mechanical strength at the time of implantation and tunable biodegradability postimplantation, sintered 45 S5 Bioglass® ceramics can breakdown and change into nanosized bone minerals under aqueous physiological conditions (Chen and Boccaccini [2006b]).

**Figure 2:**
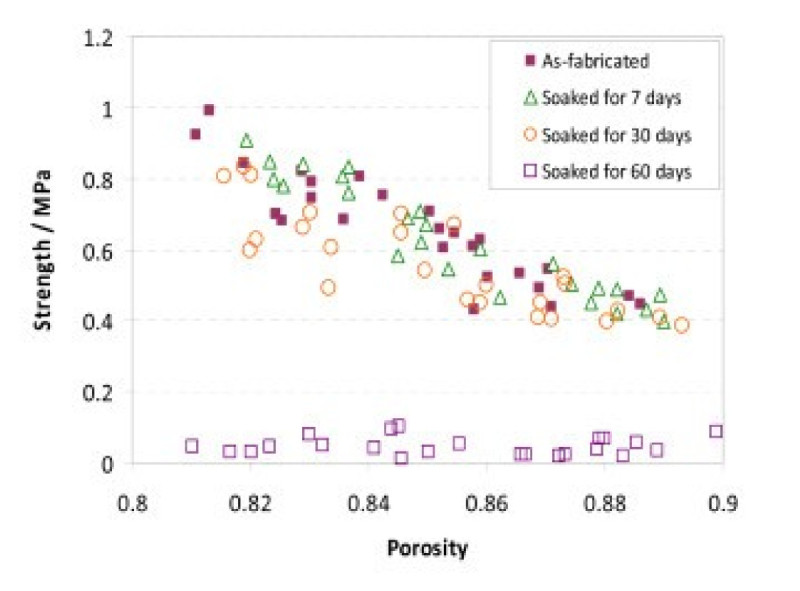
**Compressive strength of Bioglass®-PGS scaffolds.** During soaking in a tissue culture medium under physiological conditions for up to 2 months (Chen et al. [2010d]).

Our group has also has also developed a bone-like composite scaffold from PGS and 45 S5 Bioglass® (Chen et al. [2010d]). These reinforced elastomeric scaffolds have similar mechanical properties to that of cancellous bone and exhibit a mechanically steady state over prolonged periods in a physiologic environment (Figure 2). This is very relevant to engineering features in scaffolds to match the lag phase of bone repair (Chen et al. [2010d]).

## Conclusion

While the ideal tissue-engineered bone substitute should be a material, which is bioresorbable, biocompatible, and supports cell attachment, proliferation, and maturation and which is ultimately resorbed once new bone has formed, allowing this bone to undergo remodelling, this goal has yet to be achieved. To design a scaffold, it is necessary to weigh up the ‘pros and cons’ of the potential precursor materials, which are summarized in Table 7. Among the bioactive ceramics and glasses listed in Table 7, Na-containing silicon bioactive glasses offer a number of advantages. The role of silicon in biological regulation of osteogenesis and the potential to address the dilemma between mechanical strength and degradation rate make these glasses promising scaffold materials over others, such as HA and related crystalline calcium phosphates. Recent progresses on the development of borate bioactive glasses and trace element-doped bioactive glasses expand the repertoire of bioactive glasses. Although boride and other trace elements have beneficial effects on bone remodelling and/or associated angiogenesis, the risk of toxicity at high levels must be highly regarded in the design of new composition of bioactive biomaterials so that the release of these elements must be satisfactorily lower than their biologically safe levels.

**Table 7:** **Advantages and disadvantages of synthetic biomaterials used in bone tissue engineering**

Biomaterial	Advantages	Disadvantages
Calcium phosphates (e.g. HA, TCP, and biphase CaP)	(1) Excellent biocompatibility	(1) Brittle
	(2) Supporting cell activity	
	(3) Good osteoconductivity	(2) They biodegrade too slowly in the crystalline state and are mechanically too weak in the amorphous state.
Na-containing silicate bioactive glasses	(1) Excellent biocompatibility	(1) Mechanically brittle and weak at the amorphous state
	(2) Supporting cell activity	
	(3) Good osteoconductivity	
	(4) Vasculature	
	(5) Rapid gene expression	
	(6) Tailorable degradation rate	
	(7) Tailorable mechanical strength via sintering, and the issue associated with strength and degradation could be addressed	
Borate bioactive glasses	(1) Tailorable degradation rate	(1) Risk of toxicity due to the release of borate ions
	(2) Tailorable mechanical strength	
Bioactive glass ceramics (e.g., A-W)	(1) Excellent biocompatibility	(1) Brittle
	(2) Supporting cell activity	
	(3) Good osteoconductivity	(2) Slow degradation rate
Bulk biodegradable polymers		
Poly(lactic acid)	(1) Good biocompatibility	(1) Inflammation caused by acid degradation products.
	(2) Biodegradable (with a wide range of degradation rates)	
Poly(glycolic acid)	(3) Bioresorbable	
Poly(lactic-co-glycolic acid)	(4) Good processability	(2) Accelerated degradation rates cause collapse of scaffolds.
Poly(propylene fumarate)	(5) Good ductility	
Poly(polyol sebacate)	(6) Elasticity	
Surface bioerodible polymers		
Poly(ortho esters)	(1) Good biocompatibility	(1) Not completely replaced by new bone tissue
Poly(anhydrides)	(2) Retention of mechanical integrity over the degradative lifetime of the device	
Poly(phosphazene)	(3) Significantly enhanced bone ingrowth into the porous scaffolds, owing to the increment in pore size	
Composites (containing bioactive phases)	(1) Excellent biocompatibility	(1) Still not as good as natural bone matrix
	(2) Supporting cell activity	
	(3) Good osteoconductivity	
	(4) Tailorable degradation rate	(2) Fabrication techniques need to be improved.
	(5) Improved mechanical reliability	

Between the two main classes of bulk degradable and surface erodible polymer, the bulk degradable type is more promising than the surface-erosive group, considering that being replaced by new bone tissue is one of the most important criteria of an ideal scaffold material. Between thermoplastic and elastomeric polymers, Table 8 provides a comparison of both materials, as discussed earlier. Cross-linked synthetic elastomers (especially polyester elastomers) are the most attractive for use as a substitute of collagen matrix in tissue engineering. This is because, firstly, they are elastic and best match with the elasticity of biological tissue. Secondly, they are able to provide mechanical stability and structural integrity to tissues and organs without causing catastrophic mechanical implant failure, which is an issue remaining with thermoplastic rubbers. Thirdly, polyester elastomers allow closely control of structural and mechanical properties to suit various applications. Lastly and most importantly, polyester elastomers, most of which can safely breakdown to natural metabolic products by simple hydrolysis, have the potential to be tailored in their degradation rates to match healing kinetics of injured bone tissue, which can hardly achieved with current thermoplastics and thermoplastic rubbers. However, establishing the most suitable ceramic or mineral filler material and processing conditions for an elastomer is likely to provide many potential avenues for future research in bone tissue engineering scaffolds.

**Table 8:** **List of advantages and disadvantages of biodegradable polymeric biomaterials**

Material		Advantages	Disadvantages
Thermoplastic	Non-elastomers	Easy fabrication (by melt or solvent processing)	Rigid
			Lack of flexibility
		Tunable mechanical properties and degradation kinetics	Release of acidic degradation products
			Possibility of foreign body response
Elastomer	Thermoplastic	Easy fabrication	Heterogeneous degradation profile; mechanical failure; much faster than material degradation
		Flexible	
		High elongation	Release of acidic degradation products
		Tunable mechanical properties and degradation kinetics	Possibility of foreign body response
	Cross-linked	Flexible	Relatively difficult processability
		Tightly controlled purity	
		Structure, mechanical properties, and degradation kinetics	Possibility of foreign body response
		Good maintenance of form stability during degradation	Release of acidic degradation products

## Authors’ informations

QC received a Ph.D. degree in Biomaterials from Imperial College London. She is currently an academic in the Department of Materials Engineering at Monash University. Previously she was also employed by the National Heart and Lung Institute London and the University of Cambridge. She has produced more than 100 peer-refereed journal articles and book chapters. Her research interests broadly cover polymeric, ceramic, metallic, and composite materials for applications in biomedical engineering. QC acknowledges Australia Research Council Discovery Project grant: Novel artificial bone constructs for rapid vasculature and bone regeneration.

## Authors’ original submitted files for images

Below are the links to the authors’ original submitted files for images. Authors’ original file for figure 1 Authors’ original file for figure 2
