# Supplementary figures and images for: Progress and challenges in biomaterials used for bone tissue engineering: bioactive glasses and elastomeric composites
Source: Prog Biomater. 2012 Sep 26;1:2. doi: 10.1186/2194-0517-1-2 (PMC5120665; doi:10.1186/2194-0517-1-2)

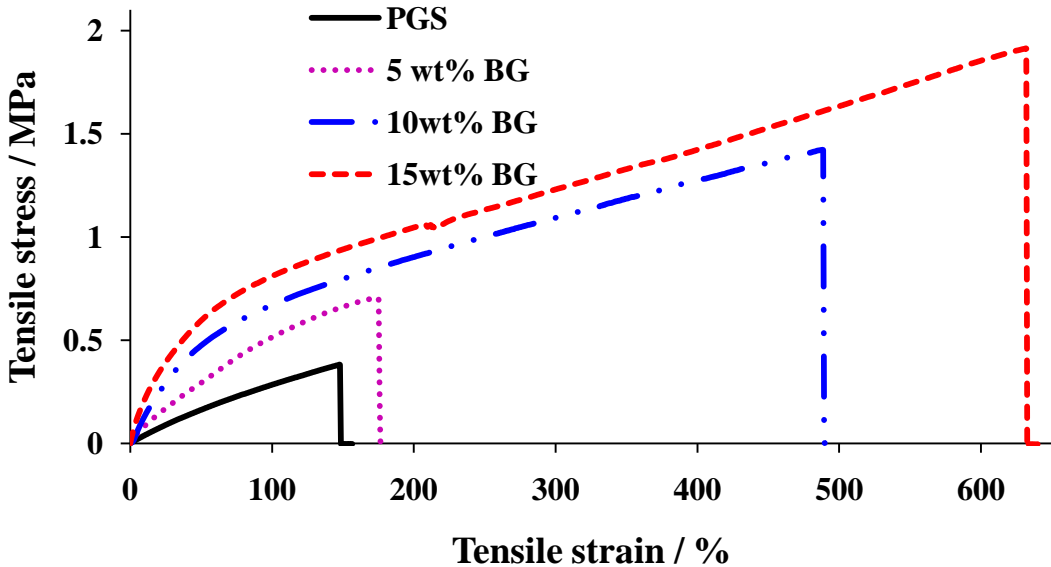

Supplement: Supplementary file 1 — Authors’ original file for figure 1 [file 40204_2012_2_MOESM1_ESM.pdf]

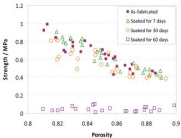

Supplement: Supplementary file 2 — Authors’ original file for figure 2 [file 40204_2012_2_MOESM2_ESM.pdf]
